# Facile Synthesis of Photofunctional Nanolayer Coatings on Titanium Substrates

**DOI:** 10.1155/2016/4318917

**Published:** 2016-03-24

**Authors:** Kyong-Hoon Choi, Jung-Gil Kim, Byungman Kang, Ho-Joong Kim, Bong Joo Park

**Affiliations:** ^1^Plasma Bioscience Research Center, Kwangwoon University, 20 Kwangwoon-gil, Nowon-gu, Seoul 139-701, Republic of Korea; ^2^Department of Electrical & Biological Physics, Kwangwoon University, 20 Kwangwoon-gil, Nowon-gu, Seoul 139-701, Republic of Korea; ^3^Nuclear Chemistry Research Division, Korea Atomic Energy Research Institute, 989-111 Daedeok-daero, Yuseong-gu, Daejeon 305-353, Republic of Korea; ^4^Department of Chemistry, Chosun University, Gwangju 501-759, Republic of Korea

## Abstract

We developed a two-step chemical bonding process using photosensitizer molecules to fabricate photofunctional nanolayer coatings on hematoporphyrin- (HP-) coated Ti substrates. In the first step, 3-aminopropyltriethoxysilane was covalently functionalized onto the surface of the Ti substrates to provide heterogeneous sites for immobilizing the HP molecules. Then, HP molecules with carboxyl groups were chemically attached to the amine-terminated nanolayer coatings via a carbodiimide coupling reaction. The microstructure and elemental and phase composition of the HP-coated Ti substrates were investigated using field-emission scanning electron microscopy and energy-dispersive X-ray spectrometry. The photophysical properties of the photofunctional nanolayer coatings were confirmed using reflectance ultraviolet-visible absorption and emission spectrophotometry. The singlet oxygen generation efficiency of the photofunctional nanolayer coatings was determined using the decomposition reaction of 1,3-diphenylisobenzofuran. The HP-coated Ti substrates exhibited good biocompatibility without any cytotoxicity, and these nanolayer coatings generated singlet oxygen, which can kill microorganisms using only visible light.

## 1. Introduction

Until recently, surgical operation using metallic implants has played an important role in orthopedics and dentistry. Among these implants, Ti and its alloys have been used extensively to fabricate implantable medical devices such as joint prostheses, facture fixation devices, and dental implants because of their excellent mechanical properties, favorable corrosion resistance, low toxicity, and biocompatibility [1–6]. Despite the advantages of these substrates, biomedical failures are still primarily caused by deficient osseointegration and implant-associated infections [7, 8]. In particular, significant complications occurring at the surface of implanted devices are mainly due to the undesirable adsorption and attachment of biomacromolecules, cells, and bacteria, ultimately resulting in foreign-body reactions and device failure [9]. Because biofilms can be very resistant to the immune response and antibiotics, it is crucial to inhibit bacterial adhesion before the formation of biofilms [10].

An effective method to prevent the formation of biofilms is to coat the surface of a Ti implant with bactericidal reagents. In the past, various antibacterial coatings have been prepared on Ti surfaces to protect patients from harmful bacteria. For instance, Hauert has already discussed the antimicrobial efficiency of Cu, Ag, and V embedded in diamond-like C films [11]. In addition, Simchi et al. reported the possibility of preventing bone implant infection using Ag-doped hydroxyapatite coatings [12]. Similar results have been reported when different materials used for antibacterial coatings are impregnated with elements such as antibiotics, quaternary ammonium compounds, iodine, fluorine, phenols, or heavy metals [13–17].

Among the alternative methods reported for pathogenic bacteria, photodynamic therapy (PDT) has been proposed as an excellent bactericidal method to combat pathogenic microbes during the past decade [18–20]. The mechanism of PDT is currently well understood. PDT combines a nontoxic PS and harmless visible light to produce reactive oxygen species that can kill cancer cell and bacterial cells. In principle, the photoactivated killing of bacteria involves the activation of a photosensitizer by irradiation with a visible light of appropriate wavelength [21]. This excited state may then undergo intersystem crossing to the slightly lower energy but longer-lived triplet state. This triplet state of the PS can either interact with oxygen to produce singlet oxygen (Type II reaction) or directly react with the molecules near the immediate vicinity (Type I reaction) [22]. Therefore, PDT is a promising alternative to treat diseases ranging from cancer to antibiotic-resistant infections because it is based on the utilization of a type of compound that inhibits or kills microorganisms using only the effect of light [23]. Therefore, technical strategy of PDT reagent coatings on implantable medical device is becoming very important because these functional materials can prevent pathogenic infections.

In a previous study, we demonstrated the preparation of a singlet-oxygen-generating nanolayer coating on a biocompatible NiTi alloy using indirect coating process [22]. In this study, we successfully fabricated hematoporphyrin- (HP-) coated Ti substrates using direct conjugation process. The photophysical and photochemical properties of the as-prepared nanolayer coatings were investigated using steady-state spectroscopy. The generation efficiency of singlet oxygen from the fabricated nanolayer coatings on Ti substrates was confirmed in a photocatalytic experiment.

## 2. Materials and Methods

### 2.1. Preparation of the HP-Coated Ti Substrate

The Ti substrates were generally polished and cleaned before functionalization. The thickness and diameter of the CP-2 Ti specimens (Seoul Titanium Inc., Korea) were 0.3 mm and 13 mm, respectively. The discs were ultrasonically cleaned sequentially with acetone, ethanol, and deionized water and then rinsed with ultrapure water. The surfaces of the Ti substrates were chemically functionalized with (3-aminopropyl)triethoxysilane (APTES) to provide active sites. Approximately 30 Ti substrates were placed into a 100 mL round flask, and 3 mL of APTES was added dropwise using a syringe. The reaction mixture was refluxed at 95°C in an oil bath with stirring for 10 h. Then, the APTES coated Ti substrates were washed thoroughly with toluene and dried at 120°C overnight to remove the toluene solvent. For the immobilization of HP via covalent bonding on the aminated Ti surface, HP (1.06 × 10^−5 ^M) was dissolved in 50 mL of ethanol. The carboxyl groups in HP were converted into active esters using 0.2 M* N*-(3-dimethylaminopropyl)-*N*′-ethylcarbodiimide (EDC) and 0.05 M* N*-hydroxysuccinimide (NHS) (for 15 min), and, then, the aminated Ti substrate was immersed in HP solution for 15 min. The HP-immobilized Ti substrate was washed thoroughly with distilled water to remove any excess reagents and dried by N_2_ blowing.

### 2.2. Characterization of the HP-Coated Ti Substrate

The surface morphology and elemental composition of the HP-coated Ti substrates were examined using a field-emission scanning electron microscope (FE-SEM, Hitachi, SU-70) equipped with an energy-dispersive X-ray spectrometer (EDS). Steady-state absorption and emission spectra were obtained using an ultraviolet-visible (UV-vis) spectrophotometer (Hitachi, U-2800) and a spectrofluorometer (Hitachi, F-4500), respectively. For the plate sample, diffuse reflectance spectra were recorded using a diffuse reflectance UV-vis spectrophotometer (Jasco V-550) equipped with an integrating sphere (Jasco ISV-469). Infrared spectra were obtained using a FT-IR spectrometer (Perkin-Elmer 100). For IR measurements, about 100 samples were prepared and then the surfaces of these samples were scraped with knife. The scraped samples were collected in an agate mortar and then prepared in the form of pressed wafers (ca. 1% sample in KBr).

### 2.3. Detection of Singlet Oxygen

The generation of singlet oxygen by the HP-coated Ti substrate was detected chemically using the 1,3-diphenylisobenzofuran (DPBF) as a reactive oxygen quencher. In a typical experiment, 3.5 mL of tetrahydrofuran (THF) solution containing the HP-coated Ti substrate and DPBF (1.0 × 10^−5 ^M) was introduced into a 1 cm quartz cell in the dark. A Xe lamp (150 W, Abet Technologies, USA) was used as the irradiation source. A 480 nm glass cut-off filter was used to filter off UV light, such that only the porphyrin Q band was irradiated, which prevents direct photodegradation of DPBF by UV light irradiation. The absorption spectra of the samples were monitored every 10 min using a UV-vis spectrophotometer.

### 2.4. Cells and Cell Cultures

A cell line, L-929 (mouse fibroblast), was obtained from the American Type Culture and Collection for the biocompatibility test and was maintained according to the provider's instructions. Briefly, the L-929 cell was cultured in minimum essential medium (MEM) supplemented with 10% fetal bovine serum (FBS) and 1% antibiotic antimycotic solution including 10,000 units of penicillin, 10 mg of streptomycin, and 25 g of amphotericin B per mL (Sigma-Aldrich Co., St. Louis, MO, USA). The cell was maintained at 37°C in a humidified atmosphere (5% CO_2_ and 95% air).

### 2.5. Biocompatibility Assessment

To confirm the biocompatibility of the HP-coated Ti substrates, cytotoxicity tests with the L-929 cell were performed, as previously described [24, 25]. Briefly, precultured L-929 cells were plated on the HP-coated Ti substrate and bare Ti substrate, which were precleaned with 70% ethanol, in a 24-well plate at 2.0 × 10^5^ cells/mL. The cells on the samples were incubated at 37°C in 5% CO_2_ for 24 h under the dark condition. After incubation, all the samples were washed three times with phosphate-buffered saline solution, and the cytotoxicity of each sample was evaluated using the cell counting kit-8 (CCK-8, Dojindo Laboratories, Kumamoto, Japan) method. The absorbance of each sample was measured at 450 nm with a multimode microplate reader (Synergy*™* HT, BioTek Instruments, Inc., VT, USA), and the relative cell viabilities are presented as a survival percentage in relation to the uncoated control.

The cells on each sample were also stained with 10 *μ*g/mL of fluorescein diacetate (FDA, Sigma-Aldrich Co., MO, USA) for 30 min to confirm the morphology of the cells, and fluorescence microscope images of the cells were obtained using an inverted fluorescence microscope (Eclipse Ti, Nikon Instruments, Inc., NY, USA) with excitation at 488 nm and emission at 530 nm.

### 2.6. Statistical Analysis

All the variables were tested in quadruplicate for each experiment, which was repeated twice (*n* = 8). The quantitative data were expressed as mean ± standard deviation. Statistical comparisons were performed using Student's* t*-test. A value of *p* < 0.05 was considered statistically significant.

## 3. Results and Discussion

**Figure 1:**
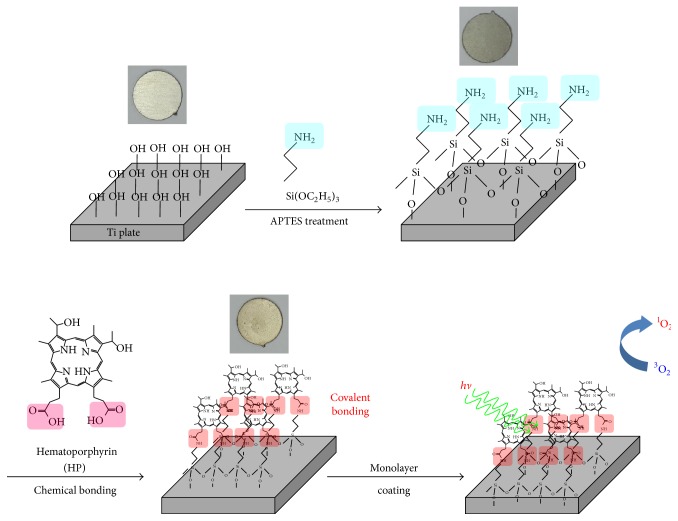
Schematic illustration of the HP-coated Ti substrate. The tetragonal photographs represent the prepared materials.

The photofunctional nanolayer coatings were fabricated by the surface modification of Ti substrates with HP molecules, which were covalently bonded to amino groups on the Ti surface, as schematically illustrated in Figure 1. In the first step, the Ti substrate was coated with APTES molecules to provide the functional amine groups. In the second step, the free terminal amino groups of APTES were covalently bonded with HP molecules via an EDC coupling reaction. As shown in tetragonal photographs of Figure 1, the color of Ti substrate is changed from silver to pale red when HP molecules were covalently bonded to surface of the Ti substrate.

**Figure 2:**
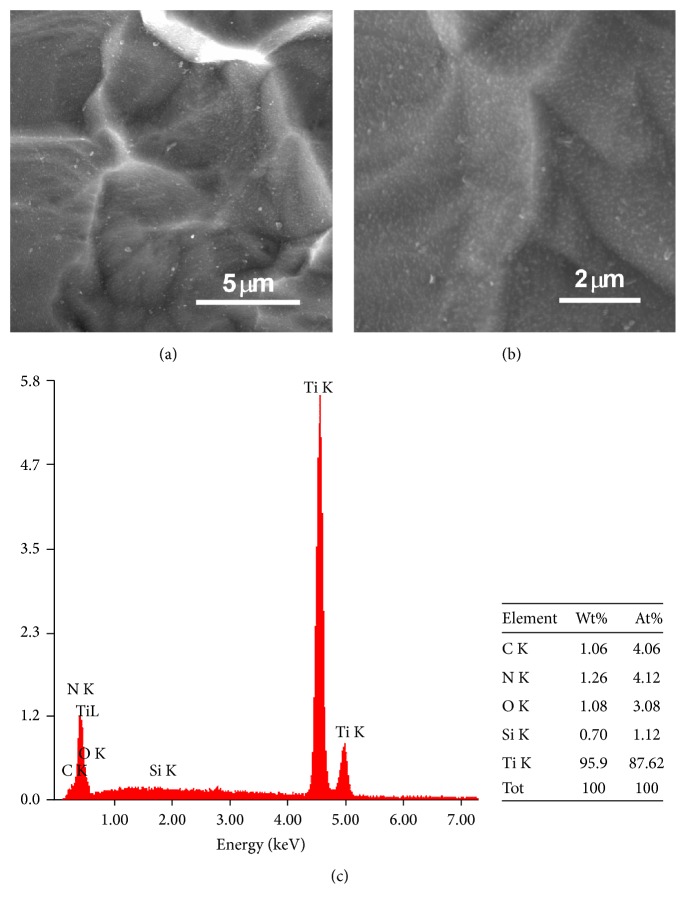
FE-SEM images ((a) and (b)) and EDS elemental analysis (c) of the HP-coated Ti substrate.

Figure 2 presents FE-SEM images and EDS analysis of the HP-coated Ti substrate. All the coating surfaces were examined using FE-SEM, and all the layers were transparent and homogeneous without any phase separation. Generally, very thin coatings have low susceptibility to thermal shock cracking and facilitate the ease of gas removal. The images of the HP-coated Ti substrate in Figures 2(a) and 2(b) reveal a rough surface with features several micrometers in length. The EDS analysis of the HP-coated Ti substrate reveals the existence of C, N, O, Si, and Ti elements, further confirming the formation of APTES and HP species on the Ti substrate (Figure 2(c)). The C, N, O, Si, and Ti element contents were 4.06, 4.12, 3.08, 1.12, and 87.62 wt%, respectively. From the Si and N contents, we can predict the APTES and HP species contents on the HP-coated Ti substrate.

**Figure 3:**
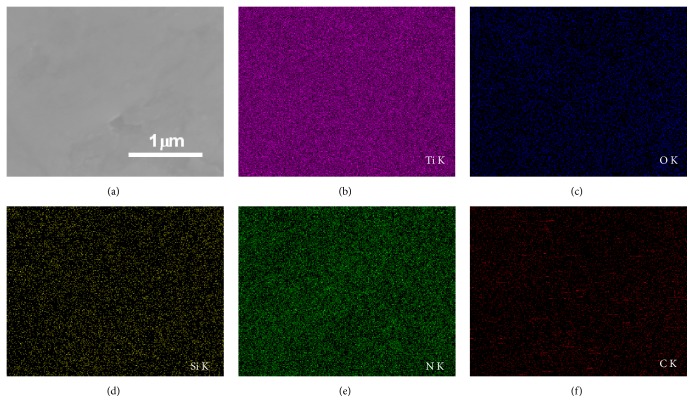
SEM image shows (a) a low-magnification image of the HP-coated Ti substrate. EDS elemental maps show the spatial distribution of (b) Ti, (c) O, (d) Si, (e) N, and (f) C in this SEM image. The spots in different colors, such as pink (Ti), blue (O), yellow (Si), green (N), and red (C), represent each element.

Figure 3 displays the micrograph and elemental X-ray maps of the HP-coated Ti substrate after the coating reaction was complete. The nanocomposite-coated Ti substrate is composed of Ti, O, Si, N, and C. The topcoat layer is mainly composed of N, C, and O, and the substrate layer is mainly composed of Ti.

**Figure 4:**
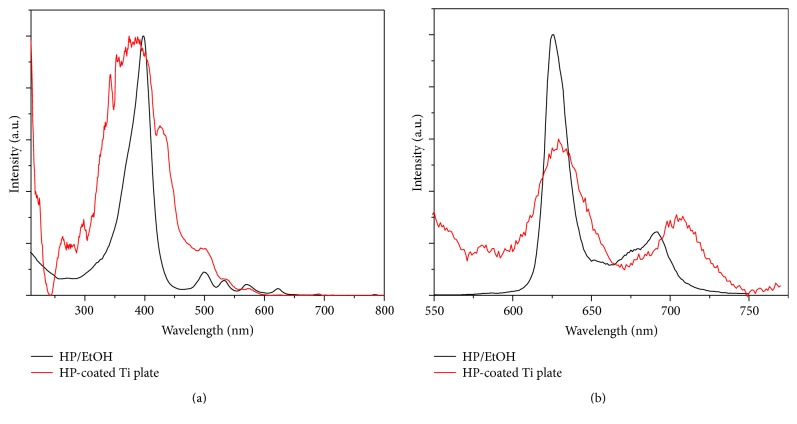
Absorption (a) and phosphorescence spectra (b) of the pure HP in ethanol and the HP-coated Ti substrate. The absorption spectrum of the HP-coated Ti substrate was obtained by applying the Kubelka-Munk function to the diffuse reflectance spectrum. The excitation wavelength was 408 nm for the emission spectrum.

UV-vis absorption and emission spectra of the pure HP and HP-coated Ti substrate are presented in Figures 4(a) and 4(b), respectively. The absorption spectrum of the HP-coated Ti substrate was measured using the diffuse reflectance UV-vis absorption method. This diffuse reflectance spectrum is translated into the absorption spectra using the Kubelka-Munk method as follows:

(1) $$\begin{array}{c} \frac{K}{S}=\frac{{\left(1-R\right)}^{2}}{2R}, \end{array}$$

where *K* represents the absorption coefficient, *S* represents the scattering coefficient, and *R* represents the absolute reflectance [26]. As observed in Figure 4(a), the absorption spectra of the pure HP in ethanol and the HP-coated Ti substrate are similar, indicating no changes in HP chromophore attachment on the Ti substrate. The phosphorescence spectrum of the HP (*λ*_ex_ = 510 nm) solution in Figure 4(b) exhibits peaks at 626 and 691 nm. However, the phosphorescence spectrum of the HP-coated Ti substrate exhibited a redshift and broadening compared with that of pure HP. The difference in the peak width and position is possibly due to the self-coupling of HP molecules bonded to the surface and the inhomogeneous bonding nature of HP molecules on the surface of the Ti substrate.

**Figure 5:**
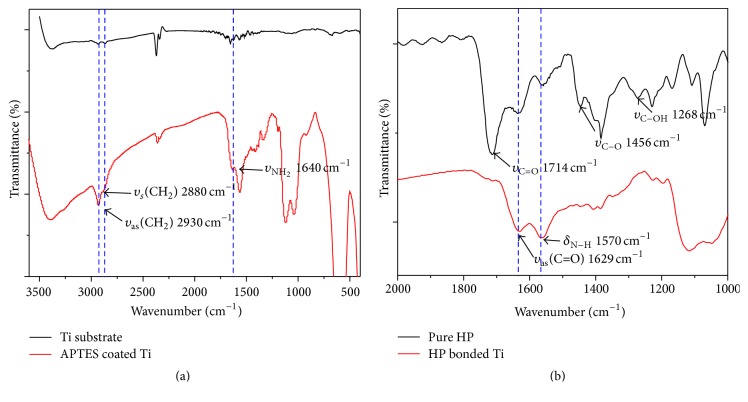
FT-IR spectra of (a) pure Ti substrate and APTES coated Ti substrate; (b) pure HP and the HP bonded Ti substrate.

To confirm the bonding between the carboxyl group of the HP and the amine group of the APTES on the surface of the Ti substrate, the FT-IR spectra were relatively investigated (Figure 5).  Figure 5(a) shows FT-IR spectra of the Ti substrate and the APTES coated Ti substrate. FT-IR spectrum of pure Ti substrate does not show some strong characteristic peaks because Ti substrate surface has no organic components. Meanwhile, IR spectrum of the APTES coated Ti substrate presents three characteristic peaks of ATPES molecules at 2930 cm^−1^ (-CH_2_ symmetric vibration mode), 2880 cm^−1^ (-CH_2_ asymmetric vibration mode), and 1640 cm^−1^ (NH_2_ stretching vibration mode), respectively, from [27]. This result indicates that the APTES molecules were coated on the Ti substrate indirectly. Also, Figure 5(b) shows FT-IR spectra of pure HP molecules and the HP bonded Ti substrate. The IR spectrum of pure HP shows major absorption peaks at 1714, 1456, and 1268 cm^−1^, corresponding to the stretching modes of the free carboxyl double bond (*υ*_C=O_), the carbon-oxygen single bond (*υ*_C-O_), and the O-H deformation (*υ*_C-OH_), respectively [28]. These absorption peak positions were caused by protonated carboxyl groups (COOH) of free HP molecules. After the conjugation reaction occurs between the carboxyl group and the amine group, IR spectrum of the HP bonded Ti substrate presents two characteristic peaks of EDC coupling reaction at 1629 cm^−1^ (C=O asymmetric stretching mode of amide I) and 1570 cm^−1^ (N-H bending mode of amide II), respectively. Therefore, this result suggests that most of the HP molecules are bound up with the surface of Ti substrate.

**Figure 6:**
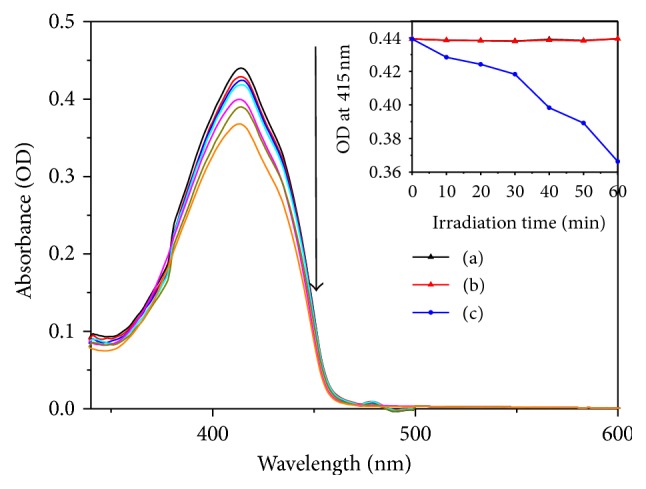
Reaction-time-dependent UV-vis spectra of DPBF in the presence of the HP-coated Ti substrate in THF solution with white light. The inset presents the decay curves of DPBF absorption OD at 415 nm as a function of irradiation duration: (a) DPBF only under light irradiation, (b) DPBF with the HP-coated Ti substrate in the dark condition, and (c) DPBF with the HP-coated Ti substrate under the light condition.

We confirmed the singlet oxygen generation by chemical decomposition using the DPBF as a singlet oxygen quencher.  Figure 6 shows the decrease in optical density (OD) at 415 nm (absorption maximum for DPBF) in the HP-coated Ti substrate as a function of the light-exposure duration. As shown in the inset of Figure 6, the ODs of the DPBF absorption peak at 415 nm are not affected by light irradiation or by the HP-coated Ti substrate without light irradiation. However, in the plot for the HP-coated Ti substrate, a gradual decrease in OD is observed with time under the light condition. This result indicates that the HP-coated Ti substrate generated a small amount of the singlet oxygen compared with the previous report [22]. However, this substrate has a very high degree of biocompatibility as it is composed of three nontoxic materials without any other additives. In addition, this substrate exhibits excellent adhesiveness as these organic components are covalently bonded to the surface of Ti. Therefore, this substrate can be used as a medical implant in antimicrobial photodynamic therapy because it generates the singlet oxygen.

To confirm the biocompatibility, we performed cytotoxicity tests of the HP-coated Ti substrate with fibroblasts in the dark condition using the recommended ISO 10993-5 method [29]. This step is critical to evaluate the possibility of its potential use in clinical fields.

**Figure 7:**
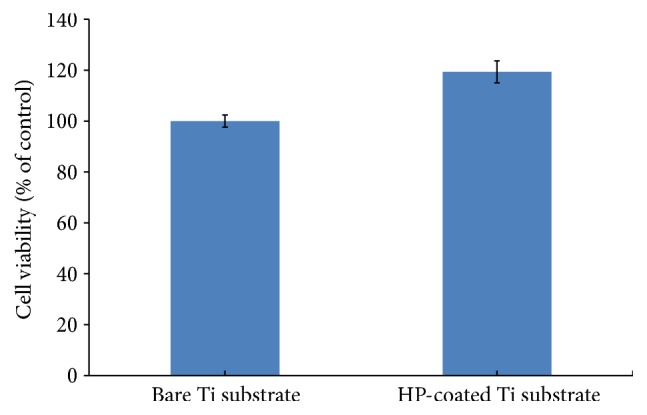
Cytotoxicity of the HP-coated Ti substrate on fibroblasts (L-929 cells). The cells were cultured on the HP-coated Ti substrate and bare Ti substrate for 24 h at 37°C in the dark condition. The data are expressed as means ± standard deviations (*n* = 8) and were analyzed using Student's* t*-tests.

Figure 7 shows that there is no cytotoxicity on the photofunctional-nanolayer-coated Ti substrates and that the photofunctionalized Ti substrates exhibit higher cell viability than the control, bare Ti substrate.

**Figure 8:**
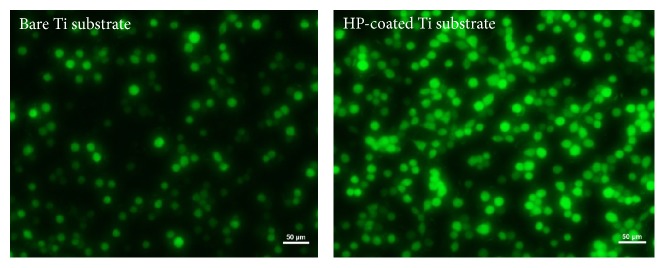
Fluorescence microscope images of cells cultured on the HP-coated Ti substrate and bare Ti substrate in the dark condition and stained with FDA. The scale bar represents 50 *μ*m.

Figure 8 presents fluorescence images stained with FDA dye for live cells after 24 h. The FDA dye passes through a living cell membrane, accumulates inside the cells, and exhibits green fluorescence excited at 488 nm; therefore, FDA can be used for detecting the cell viability [30]. The images also represent the biocompatibility of each sample and show the proportion of different cell viabilities between two samples, as demonstrated in Figure 7.

These results indicate that the HP-coated Ti substrate exhibits good biocompatibility without cytotoxicity on L-929 and can be safely applied in clinical fields.

## 4. Conclusions

In the present study, an HP-coated Ti substrate was successfully synthesized using a direct surface modification process. The surface of the HP-coated Ti substrate was rough with features several micrometers in length. The elemental X-ray maps and EDS data revealed that the topcoat layer was mainly composed of N, C, and O, indicating that the HP molecules and substrate layers were mainly composed of Ti. UV-vis and fluorescence spectra revealed that the HP molecules were covalently bonded to the amine groups on the Ti substrate directly. From the photodegradation method of DPBF, the gradual decrease of DPBF absorption depending on the irradiation time indicates the generation of singlet oxygen. These results demonstrate that the HP-coated Ti substrate has great potential to become a next-generation medical implant with antimicrobial PDT function.
